# Effects of Betulinic Acid and Ursolic Acid on IL-17-Induced CCL20 Release in Normal Human Epidermal Keratinocytes

**DOI:** 10.3390/life15071073

**Published:** 2025-07-04

**Authors:** Anna Arai, Takahiro Oyama, Toyoaki Nakajima, Michiru Usui, Ena Sato, Takanori Kamiya, Midori Oyama, Takashi Tanikawa, Tomoharu Takeuchi, Takehiko Abe, Tomomi Hatanaka

**Affiliations:** 1 Faculty of Pharmacy and Pharmaceutical Sciences, Josai University, 1-1 Keyakidai, Sakado 350-0295, Saitama, Japanoyamami@josai.ac.jp (M.O.); tanikawa@josai.ac.jp (T.T.); 2 Hinoki Shinyaku Co., Ltd., 9-6 Nibancho, Chiyoda-ku, Tokyo 102-0084, Japan; 3 School of Pharmacy, Aichi Gakuin University, 1-100 Kusumoto-cho, Chikusa-ku, Nagoya 464-8650, Aichi, Japan; takeuchi@dpc.agu.ac.jp; 4 School of Medicine, Tokai University, 143 Shimokasuya, Isehara 259-1193, Kanagawa, Japan

**Keywords:** psoriasis, epidermal keratinocyte, betulinic acid, ursolic acid, interleukin 17, C-C motif chemokine ligand 20

## Abstract

Psoriasis is a chronic inflammatory skin disease characterized by erythema, infiltration, and scaling, which is mainly caused by interleukin (IL)-17. The use of molecular targeted drugs in specific therapies offers high efficacy; however, high medical costs and a significant risk of side effects highlight the need for novel therapeutic agents. We previously observed that *Morus alba* extract (MAE) suppressed IL-17-induced *CCL20* mRNA expression in normal human epidermal keratinocytes (NHEKs). In this study, we focused on the IL-17 signaling pathway and investigated the effects of pentacyclic triterpenoids, betulinic acid (BA), and ursolic acid (UA), which are present in MAE, on NHEK cells. Real-time reverse transcription polymerase chain reaction (RT-PCR) and enzyme-linked immunosorbent assay (ELISA) revealed that both BA and UA suppressed CCL20 expression, while only UA alone inhibited CCL20 release. ELISA using specific inhibitors demonstrated that both the p38 and extracellular-signal-regulated kinase 1/2 (ERK1/2) pathways were crucial for IL-17-induced CCL20 release in NHEK. UA effectively suppressed ERK1/2 nuclear localization and moderately affected p38 phosphorylation. These results indicated that UA is a potential seed compound for psoriasis treatment through its targeting of the IL-17 pathway.

## 1. Introduction

Psoriasis is a chronic inflammatory disease characterized by excessive proliferation of skin keratinocytes [1]. In addition to the skin, up to 20% of cases develop inflammation in the joints and entheses known as psoriatic arthritis (PsA) [2]. In psoriatic lesions, interleukin (IL)-17-producing T helper (Th17) cells infiltrate and release inflammatory cytokines including IL-17 and IL-22 [3]. These promote the proliferation of keratinocytes [1], which possess receptors for these cytokines. Consequently, several substances including S100A7, IL-8, and C-C motif chemokine ligand (CCL) species are released, which promote immune cell infiltration and exacerbate inflammation [4,5]. The excessive growth of keratinocytes causes the eventual thickening, drying, and loss of barrier function in the skin [4,5]. Typical symptoms of psoriasis include erythema, skin hyperplasia, plaque formation, and itching [6]. Patients scratch the lesions, resulting in several negative effects such as skin fragility and poor appearance, reduced concentration, and sleep disturbance, which worsens the disease [7] and reduces patient quality of life. These effects are particularly prominent in young and female patients [8].

Psoriasis treatment strategies are classified into three categories: mild or localized, moderate–severe, and PsA [3]. The boundary between the first two categories was defined as the ratio of plaque lesions, which range from 3 to 10% of the total body surface area. Topical corticosteroids are the mainstay therapy for most patients with mild or localized psoriasis. Although they potently and rapidly reduce inflammation, topical corticosteroids should not be used broadly or for long-term applications due to their negative side effects. Patients who do not respond to corticosteroids are administered biologics, which specifically target psoriatic molecules such as IL-17 and IL-22. Nonetheless, the use of biologic therapies is associated with certain drawbacks, such as substantial treatment expenses, heightened susceptibility to infections resulting from immunosuppression, and restricted access to medical institutions equipped to deliver such treatments [9]. Hence, safe remedies for psoriasis that impose a low burden on patients are required.

A possible solution to this challenge may be derived from natural compounds [10,11]. Recently, we reported that *Morus alba* extract (MAE) suppressed IL-17-induced *CCL20* mRNA expression in normal human epidermal keratinocytes (NHEKs) [12]. Moreover, MAE repressed the abnormal proliferation induced by IL-17 treatment in a reconstructed 3D epidermal model. These results suggest that MAE is a potential candidate for treatment of psoriasis [12]. However, the MAE components responsible for these effects and their targets in the IL-17 pathway remain unclear.

Among the compounds reported to exist in MAE, pentacyclic triterpenoids have attracted attention for their various physiological actions, such as hepatoprotective, anti-hyperglycemia, preventative of tumor progression, and anti-inflammatory effects [13]. Therefore, the present study focused on pentacyclic triterpenoids. The most well-known pentacyclic triterpenoids in MAE are betulinic acid (BA) and ursolic acid (UA) [14,15,16,17]. BA has been reported to have immunomodulatory, antioxidant, anticancer, anti-malarial, antiviral, and anti-inflammatory effects [18,19,20,21]. Additionally, UA has been shown to have antioxidant, antiviral, hepatoprotective, anticancer, anti-angiogenesis, and anti-inflammatory effects [21,22,23,24,25]. To the best of our knowledge, however, the effects of BA and UA on IL-17-induced CCL20 expression have not yet been clarified.

Nuclear factor κB (NF-κB) [26,27,28,29,30,31,32], c-Jun N-terminal kinase (JNK) [32,33], extracellular-signal-regulated kinase 1/2 (ERK1/2) [30,31,32,33,34], and p38 mitogen-activated protein kinase (MAPK) [30,31,32,33,35] have generally been reported to act as signal transducers under the IL-17 receptor, though the specific pathways utilized vary between cell types [32]. In addition, cell type impacts regulation of the *CCL20* gene [36]. Binding sites for NF-κB, activator protein 1 (AP-1), and CCAAT/enhancer-binding protein β (C/EBPβ) have been previously observed in the promoter region of the *CCL20* gene [37,38]. To date, the signaling cascade from the IL-17 receptor to CCL20 in normal human keratinocytes has not been fully elucidated. Therefore, the present study focused on CCL20 suppression and the associated signaling pathways to investigate the effects of BA and UA on IL-17-stimulated NHEK cells.

## 2. Materials and Methods

### 2.1. Reagents

IL-17 was purchased from BioLegend Inc. (San Diego, CA, USA). Betulinic acid (BA) and ursolic acid (UA) were purchased from Tokyo Chemical Industry Co., Ltd. (Tokyo, Japan). SN50 (NF-κB inhibitor), SP600125 (JNK inhibitor), SCH772984 (ERK1/2 inhibitor), and SB202190 (p38 inhibitor) were obtained from the Cayman Chemical Company (Ann Arbor, MI, USA), BLD Pharmatech Ltd. (Shanghai, China), ChemScene LLC (Monmouth Junction, NJ, USA), and AdipoGen, Inc. (San Diego, CA, USA), respectively. BA, UA, and their inhibitors were dissolved as stock solutions in 10 mM dimethyl sulfoxide (DMSO). The antibodies used for immunoblot analyses and immunofluorescence–immunocytochemistry are listed as follows: anti-phospho-p44/42 MAPK (ERK1/2; Thr202/Tyr204) polyclonal antibody (#9101, Cell Signaling Technology Inc., Danvers, MA, USA), anti-p44/42 MAPK (ERK1/2) polyclonal antibody (#9102, Cell Signaling Technology Inc.), anti-phospho-p38 (#4511, Cell Signaling Technology Inc.), anti-p38 (#8690, Cell Signaling Technology Inc.), anti-C/EBPβ (H-7) monoclonal antibody (#sc-7962, Santa Cruz Biotechnology, Santa Cruz, CA, USA), anti-GAPDH monoclonal antibody (#60004-1-IG, Proteintech Group, Rosemont, IL, USA), anti-mouse IgG, HRP-Linked Whole Ab Sheep (NA931V, Cytiva, Marlborough, MA, USA), anti-rabbit IgG, HRP-Linked Whole Ab Donkey (NA934V, Cytiva), goat anti-rabbit IgG (H  +  L) Cross-Adsorbed Secondary Antibody Alexa Fluor™ 488 (Thermo Fisher Scientific Inc., Waltham, MA, USA), and goat anti-mouse IgG (H  +  L) Cross-Adsorbed Secondary Antibody Alexa Fluor™ 488 (Thermo Fisher Scientific Inc.).

### 2.2. Cells and Cell Culture

Primary neonatal epidermal keratinocytes derived from humans (NHEKs) were obtained from Kurabo Industries (Osaka, Japan; Lot Nos. 10338 and 11036). These cells were propagated in HuMedia-KG2, a proprietary culture solution specifically developed by Kurabo. Upon delivery, the cells were thawed and preincubated under standard culture conditions: 37 °C with 5% CO_2_ in a humidified incubator, using conventional tissue culture vessels (Sumitomo Bakelite Co., Ltd., Tokyo, Japan). For all experimental procedures, only cells maintained between 5 and 19 days post-thawing were employed. The growth medium was supplemented according to manufacturer guidelines with the following additives: 0.1 ng/mL recombinant human epidermal growth factor (EGF), 0.67 µg/mL hydrocortisone, 10 µg/mL insulin, 50 µg/mL gentamicin sulfate, 50 ng/mL amphotericin B, and 0.4% volume-to-volume bovine pituitary extract (BPE) per 500 mL of HuMedia-KG2.

### 2.3. WST-8 Cell Viability Assay

NHEK cells were plated at a density of 1.0 × 10^3^ cells per well in 96-well BioLite™ plates (Thermo Fisher Scientific Inc.). Following a 24 h preincubation period, the culture medium was exchanged for fresh medium supplemented with the specified concentrations (0–10 μM) of BA and UA. The cells were then cultured for an additional 72 h. Cell viability was assessed using the Cell Counting Kit-8 (DOJINDO Laboratories, Kumamoto, Japan) in accordance with the manufacturer’s protocol. The absorbance at 450 nm was determined using a SYNERGY H1 multimode microplate reader (Agilent Technologies Inc., Santa Clara, CA, USA).

### 2.4. Semi-Quantitative Real-Time RT-PCR

NHEK cells were seeded at 5.0 × 10^3^ cells per well in BioLite™ 96-well plates. After 24 h of preculture, the medium was replaced with fresh medium containing 0.3 μM of BA or UA and IL-17 (10 ng/mL). After 24 h of incubation, total RNA extraction and cDNA synthesis were performed using SuperPrep Cell Lysis RT Kit II (Toyobo Co., Ltd., Osaka, Japan), following the manufacturer’s instructions. Real-time reverse transcription polymerase chain reaction (RT-PCR) with SYBR Green (THUNDERBIRD^®^ SYBR qPCR Mix, Toyobo Co., Ltd.) was conducted using an Applied Biosystems StepOnePlus™ Real-Time PCR System (Thermo Fisher Scientific Inc.) with the following parameters: 95 °C for 1 min, followed by 40 cycles of denaturation at 95 °C for 10 s, and annealing/extension at 60 °C for 45 s. Variability between samples was controlled by normalization to the housekeeping gene, glyceraldehyde-3-phosphate dehydrogenase (*GAPDH*). Fold changes were calculated using the ΔΔCt method. Primers for *CCL20* and *GAPDH* were used as previously described [12]. The primer sequences were as follows: *CCL20*_Forward: GCT GCT TTG ATG TCA GTG CT; *CCL20*_Reverse: GCA GTC AAA GTT GCT TGC TG; *GAPDH*_Forward: AGC CAC ATC GCT CAG ACA C; *GAPDH*_Reverse: GCC CAA TAC GAC CAA ATC C. Amplification efficiency of all primer sets was confirmed to be within 1.0 ± 0.1.

### 2.5. Enzyme-Linked Immunosorbent Assay (ELISA)

NHEK cells were seeded at 5.0 × 10^3^ cells per well in BioLite™ 96-well plates. After 24 h of preculture, the medium was replaced with fresh medium containing 0.1 or 0.3 μM of BA or UA, and IL-17 (10 ng/mL). SN50, SP600125, SCH772984, and SB202190 were used at concentrations of 1 and 10 μM, 0.1 and 1.0 μM, 0.01 and 0.1 μM, and 0.5 and 5 μM, respectively, based on previous reports by Cho et al. [39], Cheng et al. [40], Lebedev et al. [41], and Mehta et al. [42], with cotreatment of 10 ng/mL IL-17. After 72 h of incubation, the supernatant medium was analyzed using the ELISA MAX™ Deluxe Set Human CCL20 (MIP-3α, BioLegend, Inc.), following the manufacturer’s instructions. Absorbance was measured at 450 nm using a SYNERGY H1 multimode plate reader.

### 2.6. Immunofluorescence–Immunocytochemistry (IF-IC)

NHEK cells were seeded at 2.5 × 10^4^ cells per well in an 8-well chamber slide II (AGC Techno Glass Co., Ltd., Shizuoka, Japan). After 24 h of preculture, 0.1 or 0.3 μM of BA or UA, and IL-17 (100 ng/mL) were added and incubated for 2–3 h. Chamber slides were rinsed with phosphate-buffered saline (PBS) (-) and fixed with 4% paraformaldehyde solution (FUJIFILM Wako Pure Chemical Corporation, Osaka, Japan). Cells were permeabilized with 0.2% Triton X-100 (FUJIFILM Wako Pure Chemical Corporation) in PBS(-) for 15 min at room temperature (rt, 15–25 °C). Subsequently, cells were blocked with 10% goat serum (Sigma-Aldrich, St. Louis, MO, USA) in PBS(-) for 1 h at rt. Subsequently, the samples were incubated overnight at 4 °C with a 1:100 dilution of the primary antibody in PBS(-) containing 3% goat serum. The following day, the samples were incubated with a 1:200 dilution of the secondary antibody and an appropriate dilution of Hoechst 33258 solution (DOJINDO Laboratories) in PBS(-) for 1 h at rt. Finally, the samples were mounted using Fluoro-KEEPER Antifade Reagent, Non-Hardening Type (NACALAI TESQUE, Inc., Kyoto, Japan), and covered with an NEO micro cover glass (24 × 50 mm^2^) (Matsunami Glass Ind., Ltd., Osaka, Japan). The cover glass was sealed using Can Make Colorful Nails NTC (IDA Laboratories Co., Ltd., Tokyo, Japan). Green and blue fluorescence signals were detected using the Floid Cell Imaging Station (Thermo Fisher Scientific). Quantification of green and blue image pixels was performed using Python 3 in Google Collaboratory (Google, Mountain View, CA, USA).

### 2.7. Immunoblot Analyses

NHEK cells were seeded at 2.5 × 10^4^ cells per well in 12-well plates (Sumitomo Bakelite Co., Ltd.). After 24 h of preculture, the cells were treated with 100 ng/mL IL-17 for 0–60 min. To assess the effects of BA and UA, the cells were pretreated with these compounds 30 min prior to the addition of 100 ng/mL IL-17 for 10 min. The cells were rinsed with PBS(-) and lysed with RIPA buffer (NACALAI TESQUE, Inc.) for 15 min on ice. The lysates were centrifuged at 10,000× *g* for 10 min at 4 °C to obtain the supernatant. Protein concentrations were adjusted using a TaKaRa BCA Protein Assay Kit (Takara Bio Inc., Shiga, Japan) according to the manufacturer’s instructions. Samples were mixed with Sample Buffer Solution with Reducing Reagent (6×) for Sodium dodecyl-sulfate polyacrylamide gel electrophoresis (SDS-PAGE) (NACALAI TESQUE, INC.) and loaded onto a 12.5% polyacrylamide gel (NACALAI TESQUE, INC.) at 5 µg per lane. Electrophoresis was performed at a constant current of 25 mA for 60 min. Proteins were transferred to a PVDF membrane with a 0.45 μm pore size at 50 V constant voltage for 120 min. The transfer buffer contained 20% methanol (FUJIFILM Wako Pure Chemical Corporation) in Tris-Glycine Buffer Solution (pH 8.3) (NACALAI TESQUE, Inc.). The membranes were blocked using Blocking One or Blocking One P (NACALAI TESQUE, Inc.). The primary (1:1000 dilution) and secondary (1:10,000 dilution) antibody reactions were performed with the iBind™ Flex Western System (Thermo Fisher Scientific Inc.). The membranes were washed with Tris-buffered saline (pH 7.4) containing 0.05% Tween 20 (NACALAI TESQUE, Inc.). Membranes were treated with Immobilon^®^ Crescendo or Immobilon^®^ ECL Ultra Western HRP (EMD Millipore Corporation, Darmstadt, Germany), and signals were detected using the ChemiDoc XRS Plus (Bio-Rad, Hercules, CA, USA). The bands were quantified using ImageLab software 6.1.0 build 7 (Bio-Rad).

### 2.8. HPLC Analyses

The concentrations of BA and UA in MAE (ICHIMARU PHARCOS Co., Ltd., Motosu, Japan) were quantified based on a previously reported method with minor modifications [43]. Briefly, the pentacyclic triterpenoids were separated using an HPLC system (LC-20AD, Shimadzu Corporation, Kyoto, Japan) equipped with an SPD-10A UV detector. Chromatographic separation was achieved on a reversed-phase ODS-2 column (150 mm × 4.6 mm, 5 μm particle size) maintained at 35 °C. The mobile phase consisted of acetonitrile and methanol (90:10, *v*/*v*), delivered isocratically at a flow rate of 0.5 mL/min. Prior to injection, the samples were diluted with 100% methanol. Quantification was performed using authentic chemical standards, and data were processed using the LabSolutions software 5.7.3 (Shimadzu Corporation).

### 2.9. Statistical Analysis

All quantitative data with more than duplicate measurements are presented as the mean ± standard error (SE). Statistical significance was determined by Dunnett’s test (* *p* < 0.05). Other data are shown as box plots with individual data points represented as dots. The data were normalized using the method described by Ruijter et al. [44,45]. The data were stratified by level based on the geometric means calculated for each group. The geometric means of the quotients were calculated for each level and the original data were divided by the values for each group. Statistical analyses were performed using Google Collaboratory with the runtime set to R (version 4.4.0, R Core Teams 2024). Multiple comparisons were assessed using Dunnett’s test, with the significance level set at α = 0.05.

### 2.10. Declarations of Generative AI in Scientific Writing

In composing the present manuscript, the authors employed the GPT-3.5 language model (OpenAI, San Francisco, CA, USA) for rewording and stylistic enhancement. All output generated by this system was critically reviewed and revised as necessary by the authors to ensure accuracy and appropriateness. The authors accept complete accountability for the final submitted content. Furthermore, Python-based scripts were produced using GPT-3.5 assistance during data processing and visualization.

## 3. Results

### 3.1. Effects of Betulinic Acid (BA) and Ursolic Acid (UA) on Viability of NHEK Cells

**Figure 1:**
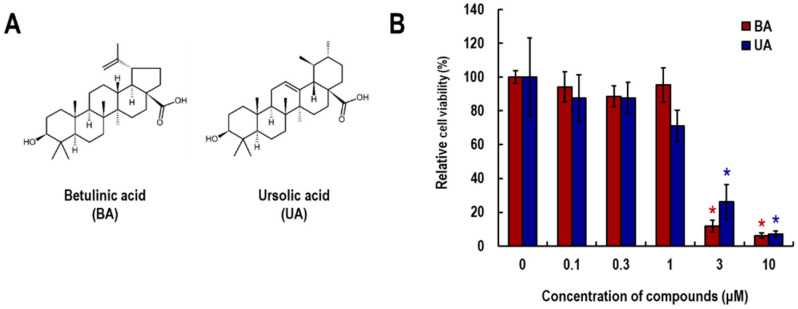
Effects of betulinic acid (BA) and ursolic acid (UA) on viability of NHEK cells. (**A**) Chemical structures of betulinic acid (BA) and ursolic acid (UA). (**B**) Cell viability was assessed using the WST-8 assay. NHEK cells were treated with 0.1, 0.3, 1, 3, and 10 μM of BA (red) and UA (blue) for 72 h. Data are presented as relative values compared to the 0.1% DMSO-treated control group (conc. 0) and shown as the mean ± SE. Statistical significance was determined by Dunnett’s test (* *p* < 0.05, *n* = 3). Each asterisk (*) is color-coded to match the corresponding treatment group (red for BA, blue for UA).

The cytotoxic effects of BA and UA (Figure 1A) were investigated using the WST-8 assay (Figure 1B). As a result, cell viability was significantly reduced by treatment with 3 and 10 μM of both compounds. In addition, UA slightly reduced cell viability at a concentration of 1 μM. In MAE, BA and UA were present at concentrations of 120.9 and 26.9 μM, respectively, suggesting that the effective dose of MAE (2.8 ppm, corresponding to a 1/2000 dilution of the commercially provided extract [12]) contains 60.5 and 13.5 nM of BA and UA, respectively (Supplementary Materials, Table S1). Based on these findings, we decided to use concentrations of both compounds up to 0.3 μM for evaluating their effects on IL-17-induced cellular responses.

### 3.2. Effects of BA and UA on IL-17-Induced CCL20 Expression and Release

**Figure 2:**
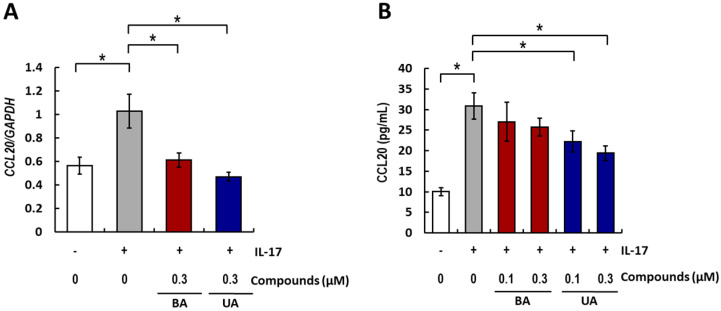
Effects of BA and UA on IL-17-induced expression and release of CCL20. Relative CCL20 mRNA expression levels (**A**) and protein release into the medium (**B**) were assessed using real-time polymerase chain reaction (RT-PCR) and enzyme-linked immunosorbent assay (ELISA), respectively. (**A**) Normal human epidermal keratinocyte (NHEK) cells were cotreated with 10 ng/mL IL-17 and 0.3 μM of betulinic acid (BA) and ursolic acid (UA) (red and blue, respectively) for 24 h. Data are presented as relative values compared to the IL-17-treated group (gray). (**B**) NHEK cells were cotreated with 10 ng/mL IL-17 and 0.1 or 0.3 μM of BA or UA (red and blue, respectively) for 72 h. The control groups (white) were treated with DMSO only in all experiments. All data are presented as the mean ± SE. Statistical significance was determined by Dunnett’s test (* *p* < 0.05, *n* = 3 to 4) compared to the IL-17-treated group (gray).

Changes in gene expression levels of IL-17-induced *CCL20* following treatment with 0.3 μM of BA and UA were assessed by RT-PCR. As shown in Figure 2A, BA and UA significantly suppressed *CCL20* expression by 37 and 52%, respectively. This assay was based on our routine screening method; therefore, the concentration tested was limited to a single point, as determined by prior cell viability assays. To further validate these findings, CCL20 protein release was measured by ELISA (Figure 2B) at two different concentrations, 0.1 and 0.3 μM. Although BA showed a moderate tendency to reduce CCL20 protein release, UA treatment significantly reduced CCL20 release in a dose-dependent manner. These results suggest that BA and UA exert some suppressive effects on IL-17-induced CCL20 expression.

### 3.3. Analyses of the Intracellular Signaling of IL-17-Induced CCL20 Expression

**Figure 3:**
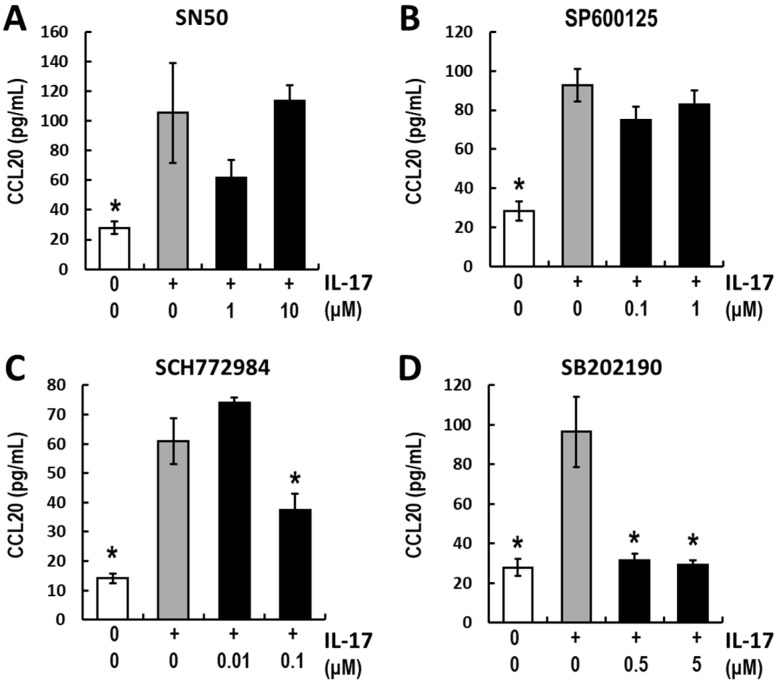
Effects of representative signaling inhibitors on IL-17-induced CCL20 release. (**A**–**D**) Normal human epidermal keratinocyte (NHEK) cells were cotreated with 10 ng/mL IL-17 and the following inhibitors (black): NF-κB inhibitor SN50 (1 and 10 μM: (**A**)), JNK inhibitor SP600125 (0.1 and 1 μM: (**B**)), ERK1/2 inhibitor SCH772984 (0.01 and 0.1 μM: (**C**)), and p38 MAPK inhibitor SB202190 (0.5 and 5 μM: (**D**)). The control groups (white) were treated with dimethyl sulfoxide (DMSO) in all experiments. All data are presented as mean ± SE. Statistical significance was determined by Dunnett’s test (* *p* < 0.05, *n* = 3 to 4) compared to the IL-17-treated group (gray).

**Figure 4:**
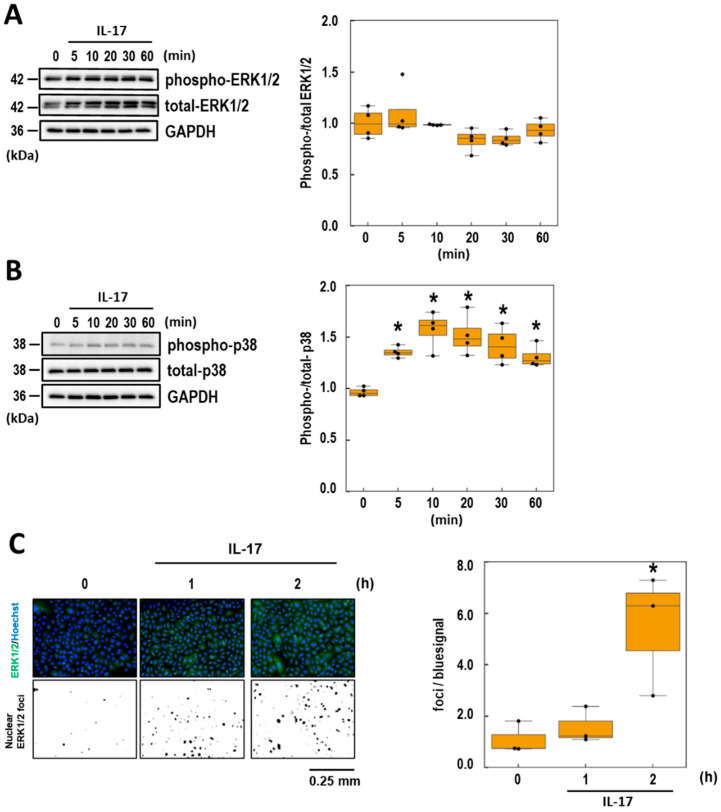
Time-dependent changes in p38 phosphorylation and ERK1/2 nuclear localization following IL-17 stimulation in normal human epidermal keratinocyte (NHEK) cells. (**A**,**B**) Immunoblot analyses of phospho-/total-ERK1/2 (**A**) and phospho-/total-p38 (**B**) after IL-17 treatment. NHEK cells were treated with 100 ng/mL IL-17 and cell lysates were collected at 0, 5, 10, 20, 30, and 60 min after treatment. Signal intensities were quantified and are presented as box plots. Phosphorylated protein levels were normalized to total protein levels. Each data point is represented as a black dot on the graph (right panels). Additionally, Glyceraldehyde 3 Phosphate Dehydrogenase (GAPDH) was detected as loading control. (**C**) Immunofluorescence images of ERK1/2 localization following IL-17 treatment. NHEK cells were treated with 100 ng/mL IL-17 for 0, 1, and 2 h. Cells were stained with green (ERK1/2) and blue (Hoechst) fluorescent dyes (upper panels). Green/blue co-localization signals were extracted and displayed in the lower panels. Quantified data of images were normalized to the non-treated group and presented as box plots (right panel). Each data point is represented as a black dot. Statistical significance was determined by Dunnett’s test (* *p* < 0.05, *n* = 3 to 4) compared to the zero-time control.

Several reports have shown that the molecules downstream of the IL-17 receptor vary depending on cell type [5,32]. Before analyzing the target pathways of BA and UA in IL-17 downstream signaling, the signaling pathways in NHEK cells were assessed. The following inhibitors targeting four major signaling molecules were selected: SN50 (NF-κB inhibitor), SP600125 (JNK inhibitor), SCH772984 (ERK1/2 inhibitor), and SB202190 (p38 inhibitor). The inhibitor concentrations were determined based on previous studies (see Materials and Methods Section 2.5). CCL20 release remained unchanged after SN50 and SP600125 treatment (Figure 3A,B). Conversely, SCH772984 and SB202190 significantly suppressed IL-17-induced CCL20 release (Figure 3C,D). These findings suggest that ERK1/2 and p38 pathways are involved in IL-17-induced CCL20 expression in NHEK cells. To further elucidate the initial signal transduction mechanism, the phosphorylation levels of ERK1/2 and p38 were assessed after 60 min of exposure to high IL-17 concentrations (100 ng/mL). ERK1/2 phosphorylation remained unchanged for at least 60 min (Figure 4A). In contrast, p38 phosphorylation levels increased rapidly and significantly within 5 min of treatment, peaking at approximately 1.5-fold after 10 min (Figure 4B). Because ERK1/2 inhibition affected IL-17-induced CCL20 expression (Figure 3C), we evaluated the nuclear localization of ERK1/2 following IL-17 treatment. The results showed a significant increase in ERK1/2 nuclear localization and co-localization with the nuclear signal (Hoechst 33342) 2 h after IL-17 treatment (Figure 4C). These data indicate that both the ERK1/2 and p38 signaling pathways are involved in IL-17-induced CCL20 release.

### 3.4. Effects of BA and UA on IL-17-Induced p38 Phosphorylation

**Figure 5:**
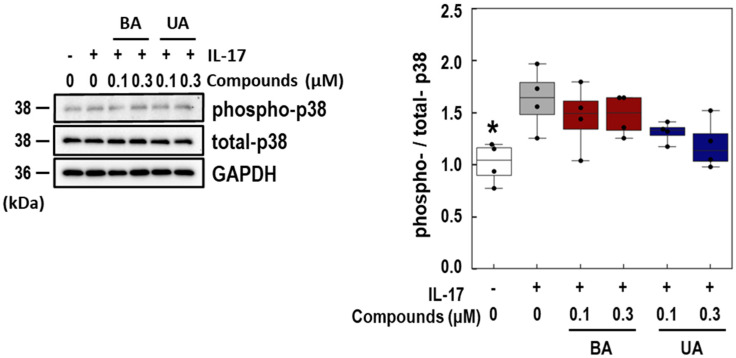
Effects of betulinic acid (BA) and ursolic acid (UA) on IL-17-induced p38 phosphorylation. Immunoblot images of phospho-/total-p38 in normal human epidermal keratinocyte (NHEK) cells treated with BA and UA in the presence of IL-17. NHEK cells were pretreated with 0.1 and 0.3 μM of BA or UA, followed by 100 ng/mL of IL-17 for 10 min. Signal intensities were quantified and are presented as box plots (right panel). Phosphorylated protein levels were normalized by the total protein levels. The BA and UA data are shown in red and blue, respectively. The control group (white) was treated with dimethyl sulfoxide (DMSO) only in the experiment. Each data point is represented as a black dot. Glyceraldehyde 3 Phosphate Dehydrogenase (GAPDH) was used as a loading control. Statistical significance was determined by Dunnett’s test (* *p* < 0.05, *n* = 4) compared to the IL-17-treated group (gray).

To investigate the target pathways of BA and UA, the phosphorylation levels of p38 in NHEK pretreated with these triterpenoids were assessed 10 min after IL-17 treatment. As shown in Figure 5, BA did not affect the phospho-p38 level induced by IL-17. Although UA slightly (*p* = 0.069) inhibited p38 phosphorylation in a dose-dependent manner, the inhibitory effect was not significant. These findings suggest that the effect of BA and UA on the p38 pathway contributes slightly to the suppression of IL-17 signaling.

### 3.5. Effects of BA and UA on IL-17-Induced ERK1/2 Nuclear Localization

**Figure 6:**
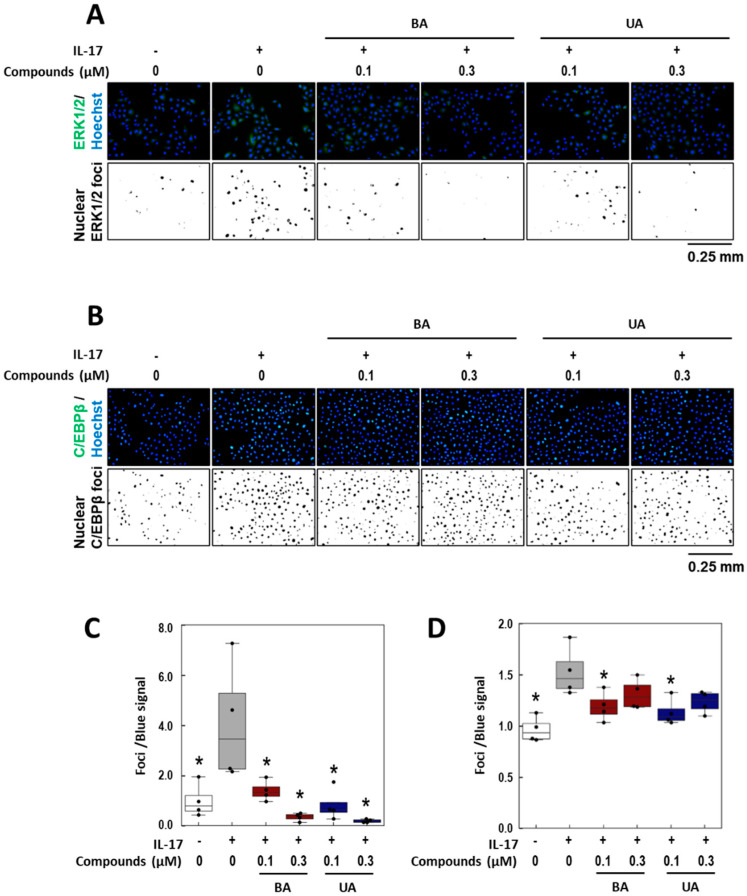
Effects of betulinic acid (BA) and ursolic acid (UA) on IL-17-induced nuclear localization of ERK1/2 and C/EBPβ. (**A**,**B**) Immunofluorescence images of ERK1/2 (**A**) and C/EBPβ (**B**) localization following treatment with IL-17 in the presence of BA and UA. Normal human epidermal keratinocyte (NHEK) cells were pretreated with 0.1 and 0.3 μM of BA or UA, followed by 100 ng/mL of IL-17 for 2 h (**A**) or 3 h (**B**). Cells were stained with green ((**A**) ERK1/2; (**B**) C/EBPβ) and blue (Hoechst) fluorescent dyes (upper panels). Green/blue co-localization signals were extracted and shown in the lower panels. Quantified data of (**A**) and (**B**) were normalized to the non-treated group and presented as box plots ((**C**) and (**D**), respectively). The control groups (white) were treated with dimethyl sulfoxide (DMSO) in all experiments. Each data point is represented as a black dot. Statistical significance was determined by Dunnett’s test (* *p* < 0.05, *n* = 4) compared to the IL-17-treated group (gray).

To examine the effects of BA and UA on IL-17-induced cellular responses, ERK1/2 nuclear localization was assessed. As shown in Figure 6A,C, nuclear co-localization signals were significantly reduced by treatment with BA and UA. Specifically, BA at 0.1 μM and 0.3 μM reduced the signals by 85.1% and 142.7%, respectively, while UA at the same concentrations reduced them by 125.8% and 151.2%, respectively. Subsequently, nuclear localization of C/EBPβ, a known downstream target of ERK1/2, was assessed using immunofluorescence microscopy. The upregulation of C/EBPβ nuclear localization by IL-17 treatment was reduced following treatment with 0.1 μM of BA or UA by 60.7 and 68.6%, respectively (Figure 6B and Figure 6D). However, treatment with both compounds at 0.3 μM attenuated this reduction. These results suggest that BA and UA suppress IL-17-induced ERK1/2 nuclear localization, but that effects on downstream molecules are limited.

## 4. Discussion

We previously identified MAE as a candidate for psoriasis treatment through its suppressive effects on IL-17-induced inflammatory responses in NHEK cells. In the present study, we investigated the effects of BA and UA-the pentacyclic triterpenoids present in MAE-on IL-17-induced CCL20 expression in NHEK cells. Within the noncytotoxic concentration range (Figure 1B), both compounds suppressed *CCL20* mRNA levels (Figure 2A), and UA suppressed IL-17-induced CCL20 protein release into the culture medium (Figure 2B). Recently, Bielecka et al. demonstrated that UA suppresses CCL20 expression induced by a cytokine mix (M5; IL-18, IL-22, IL-1α, oncostatin M, and TNF-α), all of which are related to psoriasis, in an immortal human keratinocyte cell line (HaCaT) [46]. This is consistent with our findings for NHEK cells.

**Figure 7:**
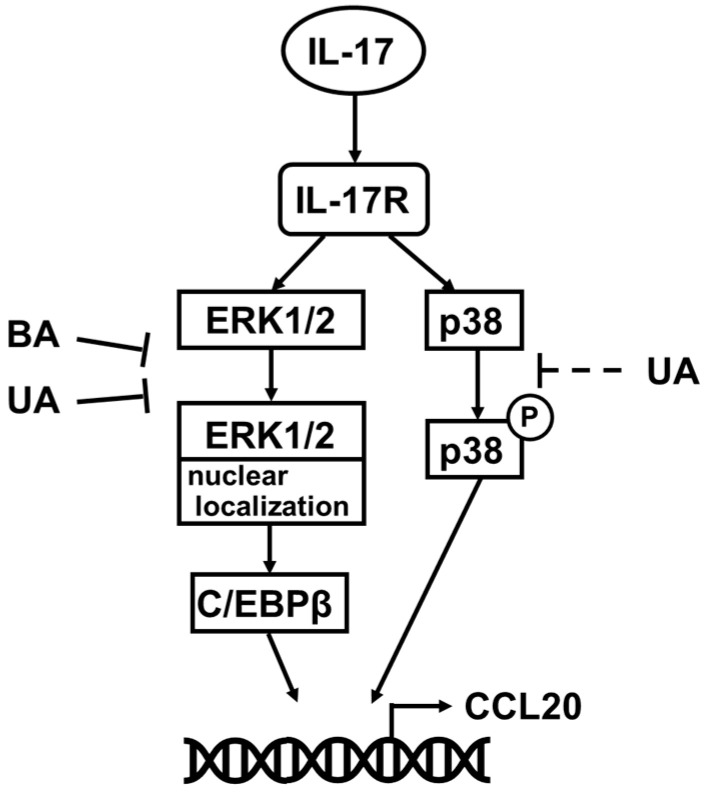
Conceptual model of the effects of betulinic acid (BA) and ursolic acid (UA) on IL-17 signaling pathways. In normal human epidermal keratinocyte (NHEK) cells, extracellular IL-17 acts on the IL-17 receptor (IL-17R). The signaling pathway downstream of IL-17R leading to CCL20 expression is broadly divided into two pathways: the p38 pathway and the ERK1/2-C/EBPβ axis. Pentacyclic triterpenoids BA and UA can significantly influence the ERK1/2-C/EBPβ axis by inhibiting ERK1/2 nuclear localization (T-shaped arrow), whereas their effect on the p38 pathway is minimal (dotted T-shaped arrow).

Several reports have addressed the downstream molecules activated by the IL-17 receptor [5,30,32]. For example, Meras et al. reported that IL-17 strongly induced CCL20 in astrocytes via the NF-κB pathway [47]. Wu et al. demonstrated that the spleen tyrosine kinase (Syk)-TNF receptor-associated factor 6 (TRAF6)-NF-κB pathway was involved in IL-17-induced CCL20 release in normal human foreskin, whereas ERK1/2 phosphorylation levels were mildly suppressed [37]. To know the target molecules of BA and UA in IL-17 downstream signaling, the effects of several inhibitors on CCL20 release from NHEK cells were assessed (Figure 3). SN50, an NF-κB inhibitor, does not reduce CCL20 release. Conversely, CCL20 release was significantly decreased by treatment with the SCH772984 and SB202190, inhibitors ERK1/2 and p38, respectively. These results are summarized in the conceptual model illustrated in Figure 7. Our findings are inconsistent with the observations mentioned above, possibly because of differences in responses to IL-17 stimuli across cell types, ethnicities, and age groups. According to the manufacturer’s instructions, the NHEK cells used in the present study were derived from neonatal Caucasian keratinocytes. Although this study includes data from two NHEK strains, direct comparisons across multiple primary cell sources were not feasible due to the limited lifespan of primary cells. Future studies should investigate signal transduction variations under psoriatic conditions using genome-wide approaches.

The presented model indicates that dual inhibition of p38 and ERK1/2 is required to suppress the IL-17 pathway in NHEK cells. UA effectively suppressed ERK1/2 nuclear localization (Figure 6A,C), but had moderate effects on p38 phosphorylation (Figure 5). These results indicated that the single use of UA alone was not suitable for blocking the IL-17 pathway. In this regard, plant extracts such as MAE warrant further investigation because they contain multiple compounds that synergistically modulate the IL-17 signaling system. Crude plant extracts have greater in vitro and/or in vivo activity than that of their isolated constituents at an equivalent dose [48]. Additionally, plant extracts are useful cosmetic ingredients that are expected to have preventive effects [49]. However, quality control for plant extract therapies is more challenging than that for synthetic compounds [50,51]. Therefore, they should be developed as an antipsoriatic agent in two ways. The first is a combination therapy with UA and other compounds, particularly those that inhibit the p38 pathway. An advantage of this strategy is that evaluating and controlling side effects is easy; however, there is a risk of unexpected side effects derived from drug–drug interactions [52]. The second method involves the synthesis of UA-based compounds that can inhibit both the ERK1/2 and p38 pathways. Single compounds designed to act on multiple targets can provide several benefits, such as more consistent pharmacokinetics, increased safety margins, greater effectiveness in complex or advanced disease states, and a reduced likelihood of resistance development [53,54]. However, the regulation of these compounds is difficult when the contribution ratios of the two targets differ [52]. Recently, Wei et al. discovered that UA inhibits sentrin-specific protease 1 (SENP1), and synthesized analogs to optimize its structure [55]. They modulated the 28-carboxyl group between the D and E rings in UA, resulting in the formation of the more potent inhibitor, No.36. Given that the synthetic strategy on the C-E rings can be applied to UA, this approach can enhance its p38 inhibitory activity, potentially leading to more effective antipsoriatic agents.

This study has several limitations. First, the direct molecular targets of BA and UA have not yet been elucidated. Although BA and UA share partial structural similarity, it remains unclear whether they bind to the same target protein and differentially regulate the ERK1/2 and p38 pathways, or act through distinct molecular targets. Further investigation is needed to clarify their precise mechanisms of action. Second, the specificity of BA and UA remains unclear. In this study, we focused only on the ERK1/2 and p38 signaling; however, other unidentified pathways may also be involved in IL-17-induced CCL20 release. Importantly, we do not intend to generalize the findings regarding CCL20 to other IL-17-responsive genes. IL-17 signaling may employ distinct downstream pathways depending on the cell type and reaction, which led us to adopt a case-by-case approach, investigating each pathway, cell type, and compound individually. This issue may be more clearly addressed by establishing pathway-specific knockout NHEK models. Although this approach was beyond the scope of the present study, we acknowledge its importance and consider it a meaningful direction for future investigation. Third, BA and UA exhibit anti-inflammatory effects in various contexts [18,19,20,21,22,23,24,25] and their known and potentially unknown targets may induce both positive and negative off-target effects on IL-17 responses in keratinocytes. Therefore, caution is warranted when considering clinical applications of these multi-target compounds. Fourth, we evaluated the dose–response relation using only two concentrations in each experiment. To more accurately define the pharmacological profile of these compounds, particularly for future therapeutic application, additional dose–response data across a broader range of concentrations would strengthen the conclusions. These limitations are currently being addressed in our ongoing research. Further preclinical investigations will be essential before drawing any therapeutic conclusions.

## 5. Conclusions

CCL20 is induced by IL-17 via the p38 and ERK1/2 pathways in NHEK cells. The pentacyclic triterpene UA strongly inhibits the ERK1/2-C/EBPβ axis within these pathways but has a weaker effect on p38 phosphorylation, highlighting the need for dual inhibition of p38 and ERK1/2 on IL-17-stimulated keratinocytes. These findings pave the way for the use of UA in the development of pentacyclic triterpene-derived synthetic compounds that specifically target psoriasis.

## Data Availability

Raw data supporting the conclusions of this article will be made available by the authors upon request.

## Supplementary Materials

The following supporting information can be downloaded at: https://www.mdpi.com/article/10.3390/life15071073/s1, Table S1: The concentrations of Betulinic acid and Ursolic acid in *Morus alba* Extracts (MAE).
