# Supplementary material for: Effects of Betulinic Acid and Ursolic Acid on IL-17-Induced CCL20 Release in Normal Human Epidermal Keratinocytes
Source: Life (Basel). 2025 Jul 4;15(7):1073. doi: 10.3390/life15071073 (PMC12298970; doi:10.3390/life15071073)
Supplement: Supplementary file 1 [file life-15-01073-s001.zip › life-3630237-supplementary.pdf]

Supplemental Table. 1

Supplemental Table S1 The concentrations of Betulinic acid and Ursolic acid in *Morus alba* Extracts (MAE).

	Betulinic acid	Ursolic acid
μg/mL	55.21	12.29
μM	120.90	26.90

The concentrations of BA and UA in MAE (ICHIMARU PHARCOS Co., Ltd, Motosu, Japan) were quantified based on a previously reported method with minor modifications [43]. Briefly, the pentacyclic triterpenoids were separated using an HPLC system (LC-20AD, Shimadzu Corporation, Kyoto, Japan) equipped with an SPD-10A UV detector. Chromatographic separation was achieved on a reversed-phase ODS-2 column (150 mm × 4.6 mm, 5 μm particle size) maintained at 35° C. The mobile phase consisted of acetonitrile and methanol (90:10, v/v), delivered isocratically at a flow rate of 0.5 mL/min. Prior to injection, the samples were diluted with 100% methanol. Quantification was performed using authentic chemical standards, and data were processed using the LabSolutions software (Shimadzu Corporation).

43.Taralkar S. V., Chattopadhyay S. A HPLC Method for Determination of Ursolic Acid and Betulinic Acids from their Methanolic Extracts of Vitex Negundo Linn. Journal of Analytical & Bioanalytical Techniques 2012, 3, 1–6. DOI:10.4172/2155-9872.1000134.

whole membranes

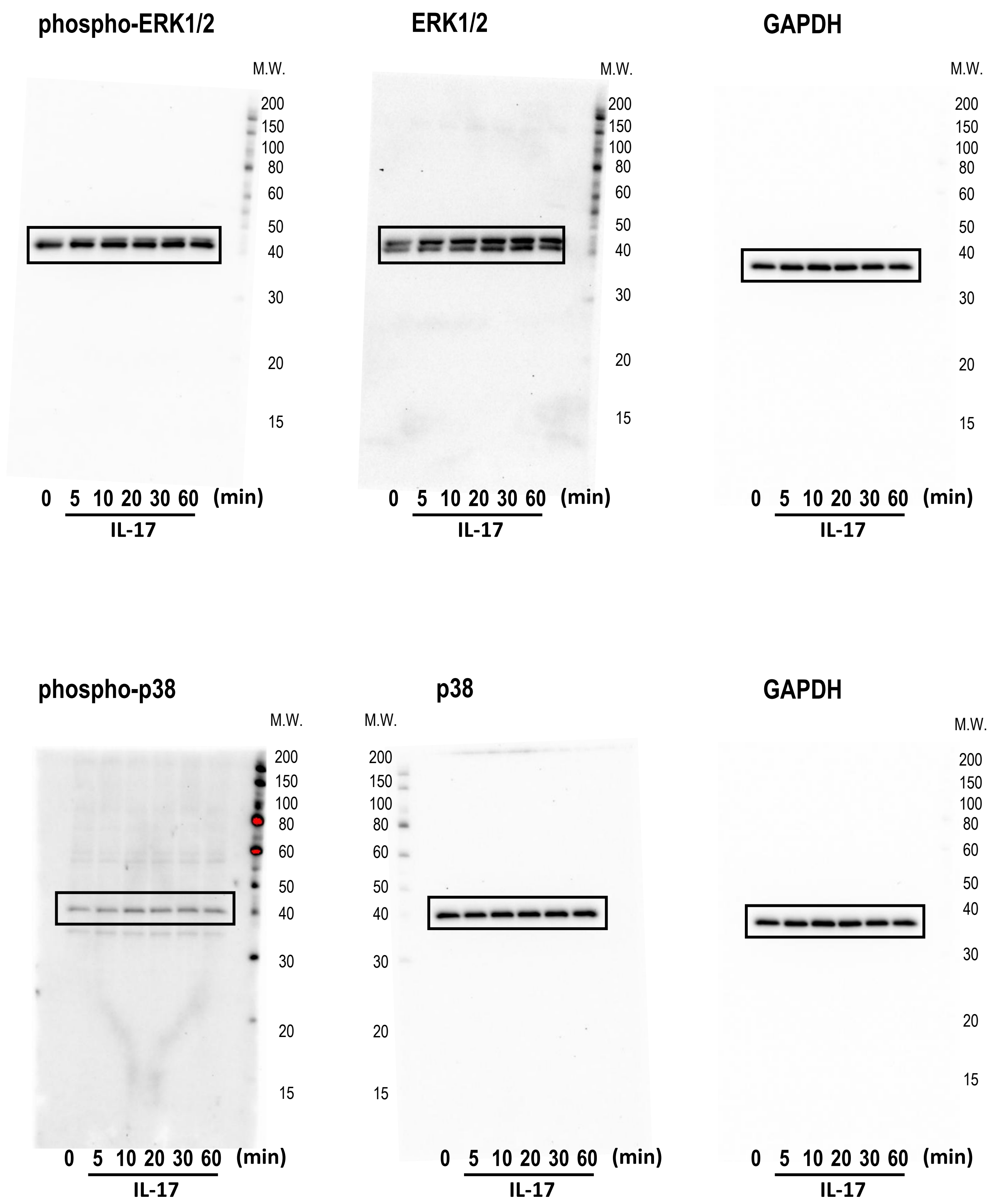

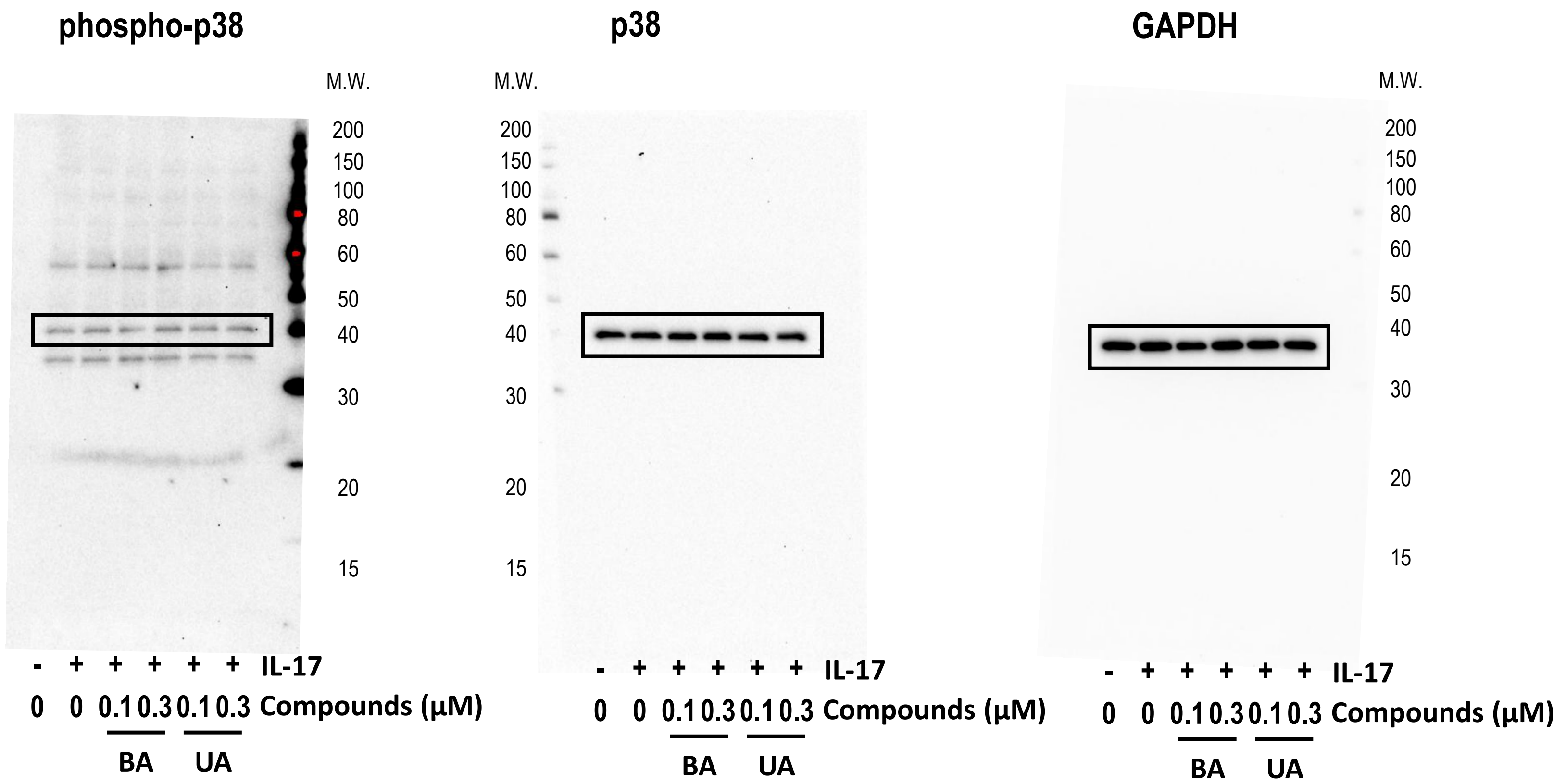
phospho-p38
M.W.
200
150
100
80
60
50
40
30
20
15
- + + + + + IL-17
0 0 0.1 0.3 0.1 0.3 Compounds (μM)
BA UA
p38
M.W.
200
150
100
80
60
50
40
30
20
15
- + + + + + IL-17
0 0 0.1 0.3 0.1 0.3 Compounds (μM)
BA UA
GAPDH
M.W.
200
150
100
80
60
50
40
30
20
15
- + + + + + IL-17
0 0 0.1 0.3 0.1 0.3 Compounds (μM)
BA UA
